# Recombinant adenoviruses expressing HPV16/18 E7 upregulate the HDAC6 and DNMT3B genes in C33A cells

**DOI:** 10.3389/fcimb.2024.1459572

**Published:** 2024-10-01

**Authors:** Yunting Shao, Pir Tariq Shah, Qisheng Su, Shanhu Li, Fang Huang, Jun Wang, Peng Wang, Chengjun Wu

**Affiliations:** ^1^ Faculty of Medicine, School of Basic Medical Sciences, Dalian University of Technology, Dalian, China; ^2^ Department of Cell Engineering, Beijing Institute of Biotechnology, Beijing, China; ^3^ Obstetrics and Gynecology Department, The Second Affiliated Hospital of Dalian Medical University Mailing, Dalian, China

**Keywords:** high-risk HPV, recombinant virus, E6/E7 gene, HDAC6, DNMT3B

## Abstract

**Objective:**

High-risk human papillomavirus (HPV) is a carcinogenic virus associated with nearly all cases of cervical cancer, as well as an increasing number of anal and oral cancers. The two carcinogenic proteins of HPV, E6 and E7, can immortalize keratinocytes and are essential for HPV-related cellular transformation. Currently, the global regulatory effects of these oncogenic proteins on the host proteome are not fully understood, and further exploration of the functions and carcinogenic mechanisms of E6 and E7 proteins is needed.

**Methods:**

We used a previously established platform in our laboratory for constructing recombinant adenoviral plasmids expressing the HPV16 E7 gene to further construct recombinant virus particles expressing HPV16/18 E6, E7, and both E6 and E7 genes. These recombinant viruses were used to infect C33A cells to achieve sustained expression of the HPV16/18 E6/E7 genes. Subsequently, total RNA was extracted and RNA-Seq technology was employed for transcriptome sequencing to identify differentially expressed genes associated with HPV infection in cervical cancer.

**Results:**

RNA-Seq analysis revealed that overexpression of the HPV16/18 E6/E7 genes upregulated GP6, CD36, HDAC6, ESPL1, and DNMT3B among the differentially expressed genes (DEGs) associated with cervical cancer. Spearman correlation analysis revealed a statistically significant correlation between the HDAC6 and DNMT3B genes and key pathways, including DNA replication, tumor proliferation signature, G2M checkpoint, p53 pathways, and PI3K/AKT/mTOR signaling pathways. Further, qRT-PCR and Western blot analyses indicated that both HPV16/18 E7 can upregulate the expression of HDAC6 and DNMT3B, genes associated with HPV infection-related cervical cancer.

**Conclusion:**

The successful expression of HPV16/18 E6/E7 in cells indicates that the recombinant viruses retain the replication and infection capabilities of Ad4. Furthermore, the recombinant viruses expressing HPV16/18 E7 can upregulate the HDAC6 and DNMT3B genes involved in cervical cancer pathways, thereby influencing the cell cycle. Additionally, HDAC6 and DNMT3B are emerging as important therapeutic targets for cancer. This study lays the foundation for further exploration of the oncogenic mechanisms of HPV E6/E7 and may provide new directions for the treatment of HPV-related cancers.

## 1 Introduction

Human papillomavirus (HPV) infection is a major global concern, causing critical public health issues worldwide (Hastings et al., 2017). As the most common sexually transmitted virus, HPV infects millions of people and leads to over 600,000 HPV-related malignancies each year (Pellom et al., 2021). HPV is one of the most potent carcinogens and has been linked to multiple types of cancers (Moeinzadeh et al., 2020), including 95% of cervical cancers, 88% of anal cancers, as well as 70% of vaginal cancers, 50% of penile cancers, 43% of vulvar cancers, and 26% of oropharyngeal cancers (Mahumud et al., 2019). Persistent infection with high-risk HPV (HR-HPV), most commonly HPV16 and HPV18, is a leading cause of cervical cancer (Takahashi et al., 2022). HPV DNA is detected in 99.7% of cervical cancers and the majority of other anogenital and head and neck cancers (Wan et al., 2022). HPV-induced cancers are addicted to the expression of the major viral oncogenes, E6 and E7, whose combined effect is required for the development and maintenance of the transformed phenotype (Hoppe-Seyler et al., 2018).

E6 and E7 are highly effective in blocking cell cycle negative regulators (Matijevic et al., 2011). This prevents the cells from maturing, causing them to continuously engage in the cell cycle and stop undergoing apoptosis. The overall result of expressing E6 and E7 is the stimulation of the cell cycle (driven by E7) and the inability of the cell to recognize this abnormal proliferation (driven by E6) (Das et al., 2020). The resulting genetic instability leads to the continuous accumulation of genetic variations, ultimately transforming the HPV-infected cells into invasive cancer cells. Additionally, in keratinocytes expressing HPV16 E6 and E7, proteins associated with cell organization and differentiation are significantly downregulated (Bhat et al., 2022). It is well known that the LXCXE motif of E7 binds to the pRb tumor suppressor, inducing pRb degradation, thereby promoting cell proliferation and facilitating HPV DNA amplification (White, 2019). E6 targets p53 for degradation, evading p53-dependent apoptosis, promoting cell survival, and completing the viral life cycle (Chen et al., 1996). Abrogation of E6 and E7 expression in tumors, or in cell-lines derived from tumors, results in growth arrest and the rapid death of the tumor cell by apoptosis or senescence, making E6 and E7 ideal potential targets for therapeutic intervention in HPV-induced cancers.

High-risk HPV E6 and E7 oncoproteins are essential for the transformation of host cells; however, the mechanisms by which they disrupt cellular protein regulation have not been fully elucidated (Bienkowska-Haba et al., 2018). Existing HPV vaccines can prevent HPV infections, but they are ineffective against existing HPV infections and cannot inhibit the progression of cancer or malignant tumors (Padash Barmchi et al., 2021). Additionally, there are currently no virus-specific therapies for cancers related to HPV infection, and the effectiveness of standard surgical and chemo/radiotherapy treatments for these cancers is very limited (Wendel Naumann and Leath, 2020; Zhuang et al., 2020). It is well known that the expression of the two viral oncogenes, E6 and E7, is crucial for inducing and maintaining the transformed phenotype of cells; still, their detailed oncogenic mechanisms have not been fully elucidated (Gao et al., 2011). Notably, HPV16 E6 and E7, as non-cellular oncoproteins, are not expressed in normal cells, and the isolation and culturing of HPV *in vitro* are extremely difficult, thus limiting the study of the oncogenic mechanisms of E6 and E7 (Zhu et al., 2021; Wang et al., 2022).

In this study, we utilized a successfully established platform for constructing recombinant adenovirus vectors expressing the HPV16 E7 gene to further construct recombinant virus particles expressing HPV16/18 E6 alone, HPV18 E7 alone, and both HPV16 E6 and E7, and HPV18 E6 and E7 simultaneously. These recombinant viruses were used to infect cells to achieve sustained expression of the HPV16/18 E6/E7 genes in the cells. Subsequently, total RNA was extracted from the cells and subjected to RNA-Seq analysis. Bioinformatics analysis was then conducted to identify significantly differentially expressed genes (DEGs) related to HPV infection and the signaling pathways they are involved in. Through cross-screening of HPV infection-related DEGs and cervical cancer-related DEGs, we identified five significantly upregulated DEGs, e.g., GP6, CD36, HDAC6, ESPL1, and DNMT3B.

Among them, HDAC6, a histone deacetylase, has become an important therapeutic target for cancer (Yu et al., 2017). In the KEGG Viral Carcinogenesis - Reference Pathway, we found that in the cell transformation pathways associated with HPV-related cervical cancer, anal cancer, penile cancer, and head and neck cancer, E7 directly regulates the HDAC6 gene. DNMT3B, a DNA methyltransferase, is responsible for *de novo* DNA methylation and plays a crucial role as a potential prognostic biomarker and molecular therapeutic target in head and neck squamous cell carcinoma (HNSCC) (Yamaguchi et al., 2017; Goričan et al., 2023). Additionally, HPV16/18 infection is one of the most common risk factors for HNSCC, and the expression of DNMT3B is also influenced by HPV infection (Sen et al., 2017; Chen et al., 2018; Heawchaiyaphum et al., 2021). Furthermore, studies have observed altered levels of DNA methyltransferases in HPV-related tumors and cell lines (Su et al., 2013; Minarovits et al., 2016). In particular, research has found significant overexpression of DNMT3B mRNA transcripts in cervical cancer cell lines Hela and Caski (Su et al., 2013). In this study, qRT-PCR and Western blot results further confirmed that the recombinant viruses expressing HPV16/18 E7 can upregulate the expression of HDAC6 and DNMT3B, thereby affecting the cell transformation process. This study lays the foundation for further exploration of the carcinogenic mechanisms of HPV E6/E7 and may provide new directions for researching the treatment of HPV infection-related cancers.

## 2 Materials and methods

### 2.1 Bacterial strains, plasmids, and cell culture

All *Escherichia coli* strains and plasmids used in this study are listed in **Supplementary Table S1**. *Escherichia coli* strains were grown in LB medium at 30°C and selected with appropriate antibiotics [chloramphenicol (Cm), 10 µg/ml; ampicillin (Amp), 10 µg/ml; kanamycin (Kan), 10 µg/ml and tetracycline (Tet), 34 µg/ml]. The concentration of 10% L-arabinose used for induction was 25 mg/ml. C33A, HEK293T, Siha, Hela and Caski cells were preserved in our laboratory. The cells were maintained in DMEM (EallBio, China) or MEM, RPMI1640 supplemented with 10% FBS and 1% penicillin-streptomycin (P/S) and incubated at 37 °C with 5% humidified CO_2_.

### 2.2 Generation of recombinant viruses expressing HPV16/18 E7

#### 2.2.1 Construction of recombinant HPV16/18 E6/E7 expression virus plasmids

The process involved digesting the intermediate plasmid pGGA-Ad4NPR-C16E7P to obtain a vector fragment containing Ad4 E1A homologous arms. Subsequently, the HPV16 E6, HPV16 E6/E7, HPV18E6, HPV18E7, and HPV18 E6/E7 genes were ligated individually to these vector fragments using T4 DNA ligase. This resulted in the creation of intermediate plasmids pGGA-Ad4NPR-C16E6P, pGGA-Ad4NPR-C16E6E7P, pGGA-Ad4NPR-C18E6P, pGGA-Ad4NPR-C18E7P, and pGGA-Ad4NPR-C18E6E7P. The constructs and components of the intermediate plasmids are shown in **Table 1**.

**Table 1:** Constructs and components of intermediate plasmids.

Target genes	Vector fragments	Intermediate plasmids
HPV16 E6	pGGA-Ad4NPR	pGGA-Ad4NPR-C16E6P
HPV16 E6/E7	pGGA-Ad4NPR	pGGA-Ad4NPR-C16E6E7P
HPV18E6	pGGA-Ad4NPR	pGGA-Ad4NPR-C18E6P
HPV18E7	pGGA-Ad4NPR	pGGA-Ad4NPR-C18E7P
HPV18 E6/E7	pGGA-Ad4NPR	pGGA-Ad4NPR-C18E6E7P

The HPV16 E6, E6/E7 and HPV18 E6, E7, E6/E7 genes with Ad4 E1A homologous arms were amplified by PCR from the intermediate plasmid to serve as the recombinant target fragments. Subsequently, the recombinant ccdB-KanPS expression vector, pBR322-Ad4-E1Amut-ccdBKanPS, was digested with restriction enzymes. The ccdB positive and negative selection system was then used in conjunction with the ExoCET recombination system to construct recombinant HPV16/18 E6/E7 adenovirus plasmids, including pBR322-Ad4-E1Amut-C16E6P, pBR322-Ad4-E1Amut-C16E6E7P, pBR322-Ad4-E1Amut-C18E6P, pBR322-Ad4-E1Amut-C18E7P, and pBR322-Ad4-E1Amut-C18E6E7P. The constructs and components of recombinant HPV16/18 E6/E7 expression virus plasmids are shown in **Table 2**.

**Table 2:** Constructs and components of recombinant HPV16/18 E6/E7 expression virus plasmids.

Target genes	Vector fragments	Recombinant adenovirus plasmids
C16E6P	pBR322-Ad4-E1Amut-ccdBKanPS	pBR322-Ad4-E1Amut-C16E6P
C16E6E7P	pBR322-Ad4-E1Amut-ccdBKanPS	pBR322-Ad4-E1Amut-C16E6E7P
C18E6P	pBR322-Ad4-E1Amut-ccdBKanPS	pBR322-Ad4-E1Amut-C18E6P
C18E7P	pBR322-Ad4-E1Amut-ccdBKanPS	pBR322-Ad4-E1Amut-C18E7P
C18E6E7P	pBR322-Ad4-E1Amut-ccdBKanPS	pBR322-Ad4-E1Amut-C18E6E7P

#### 2.2.2 Generation of recombinant virus particles

The recombinant virus genomes pAd4-E1Amut-C16E6P, pAd4-E1Amut-C16E6E7P, pAd4-E1Amut-C18E6P, pAd4-E1Amut-C18E7P, and pAd4-E1Amut-C18E6E7P were released by restriction enzyme digestion. Lipo-3000 (ThermoFisher, USA) was used to transfect the recombinant virus genomes into HEK293T cells to produce viral particles. The obtained medium containing recombinant viruses was then used to infect HEK293T cells for virus amplification.

### 2.3 qRT-PCR analysis

To assess the transcript levels of the E6/E7 gene of these obtained recombinant adenoviruses, the C33A cells were infected with the six recombinant viruses Ad4-HPV16E6, Ad4-HPV16E7, Ad4-HPV16E6E7, Ad4-HPV18E6, Ad4-HPV18E7, and Ad4-HPV18E6E7 for 24 hours. C33A cells infected with Ad4 were used as the control group. Subsequently, the RNA of the cells was collected and subjected to qRT-PCR to determine the levels of E6/E7 gene transcripts. Primers for qRT-PCR detection of HPV16 E6 were 16E6-F (forward primer, 5-AGGAGGAGGATGAAATAGATGG-3) and 16E6-R (reverse primer, 5-GCACAACCGAAGCGTAGA-3); Primers for qRT-PCR detection of HPV16 E7 were 16E7-F (forward primer, 5-AGGAGGAGGATGAAATAGATGG-3) and 16E7-R (reverse primer, 5-GCACAACCGAAGCGTAGA-3); Primers for qRT-PCR detection of HPV18 E6 were 16E7-F (forward primer, 5-AGGAGGAGGATGAAATAGATGG-3) and 18E6-R (reverse primer, 5-GCACAACCGAAGCGTAGA-3); Primers for qRT-PCR detection of HPV18 E7 were 18E7-F (forward primer, 5-AGGAGGAGGATGAAATAGATGG-3) and 18E7-R (reverse primer, 5-GCACAACCGAAGCGTAGA-3); Primers for qRT-PCR detection of GAPDH were GAPDH-F (forward primer, 5- GGAAGGTGAAGGTCGGAGTC -3) and GAPDH-R (reverse primer, 5- GAAGGGGTCATTGATGGCAAC -3).

### 2.4 RNA-seq sample collection and preparation

#### 2.4.1 RNA quantification and qualification

The C33A cells were subjected to infection with the recombinant viruses Ad4-HPV16/18 E6, E7, E6/E7 and Ad4 (control group) for a duration of 24 hours. A blank control group was also included, which did not undergo viral infection. Total RNA was extracted from all groups using Trizol Reagent (Thermo Fisher Scientific). The RNA Nano 6000 Assay Kit of the Bioanalyzer 2100 system (Agilent Technologies, CA, USA) was employed to assess the quantity and quality of RNA.

#### 2.4.2 Library preparation for transcriptome sequencing

The RNA sample preparations utilized total RNA as the input material. The PCR product was purified using AMPure XP beads, and the library was ultimately obtained via PCR amplification. Following the construction of the library, it was initially assessed using the Qubit2.0 Fluorometer, subsequently diluted to 1.5ng/ul, and the insert size was determined using the Agilent 2100 bioanalyzer. Upon meeting the expected insert size, qRT-PCR was employed to precisely quantify the effective concentration of the library, ensuring that it surpasses 2nM and meets the requisite quality standards.

#### 2.4.3 Clustering and sequencing

The samples were subsequently sequenced using the Illumina NovaSeq 6000 platform, generating paired-end reads of 150 bp. The sequenced flow cell underwent the addition of four fluorescently labeled dNTPs, DNA polymerase, and splice primers, followed by amplification. The sequencer captured the fluorescence signal and converted it into a sequencing peak using computer software, enabling the acquisition of sequence information for the fragment under examination.

### 2.5 RNA-seq data analysis

#### 2.5.1 Permission to reuse and copyright

The reference genome and gene model annotation files were obtained directly from the genome website (http://www.ncbi.nlm.nih.gov/genome/genomes/14095). The reference genome was indexed using Hisat2 (v2.0.5), and the paired-end clean reads were aligned using the same tool. The selection of Hisat2 as the mapping tool was based on its ability to generate a splice junction database from the gene model annotation file, resulting in superior mapping outcomes compared to non-splice mapping tools.

#### 2.5.2 Quantification of gene expression level

The quantification of gene expression levels was performed using feature Counts (v1.5.0-p3) to enumerate the number of reads mapped to each of the two genes. Subsequently, the Fragments Per Kilobase of transcript sequence per million base pairs sequenced (FPKM) were computed for each gene, taking into account both the gene length and the mapped reads count. FPKM is a widely adopted method for estimating gene expression levels, as it accounts for the impact of sequencing depth and gene length on the reads count (Yan et al., 2018).

#### 2.5.3 Differential expression analysis

The DESeq2 R package (version 1.20.0) was utilized to conduct the differential expression analysis between two conditions/groups, each with two biological replicates. DESeq2 employs statistical routines based on the negative binomial distribution to determine differential expression in digital gene expression data. The resulting p-values were subjected to Benjamini and Hochberg’s approach for controlling the false discovery rate. Significantly differential expression was defined as padj<=0.05 and |log2(foldchange)| >=1, which served as the threshold. Intersecting sets of differentially expressed genes were visualized with UpSet diagrams and log2 fold changes were visualized using the pheatmap package (version 1.0.12) in R (version 3.6.2).

#### 2.5.4 Enrichment analysis of differentially expressed genes

The KEGG database serves as a valuable resource for comprehending the overarching functionalities and utilities of biological systems, encompassing the cell, organism, and ecosystem, through the analysis of molecular-level data, particularly extensive molecular datasets produced by genome sequencing and other advanced experimental methodologies (http://www.genome.jp/kegg/). The integration of human disease-related genes is facilitated by the DisGeNET database. The statistical enrichment of differential expression genes in KEGG and DisGeNET pathways was assessed using the clusterProfiler R package (version 3.8.1). P<0.05 was considered to indicate a statistically significant difference.

### 2.6 Protein–protein interactions network

The STRING database (http://string-db.org) aims to provide a comprehensive assessment and integration of PPIs, encompassing both indirect (functional) and direct (physical) associations (Li et al., 2020). To evaluate the interactive relationship among differentially expressed genes (DEGs), the DEGs were first mapped to STRING. All associations obtained in STRING were provided with a confidence score. Only experimentally validated interactions with a combined score >0.4 were considered as significant. Subsequently, the PPI network was constructed using the Cytoscape software (Ver. 3.7.1). The plug-in Molecular Complex Detection (MCODE) was employed to screen the modules of PPI network in Cytoscape. PPI enrichment p-value<0.05 was considered to indicate a statistically significant difference.

### 2.7 Statistical analysis

All the experiments were conducted for three independent repeats. Statistical analysis was conducted using GraphPad Prism software (GraphPad Software). Data are presented as the mean ± SD. The t-test or One-Way Analysis of Variance (ANOVA) was performed in order to determine significant differences in measured variables among groups. The correlation between genes and pathway scores was analyzed by Spearman correlation. The p-value <0.05 was considered statistically significant.

## 3 Results

### 3.1 Recombinant viruses expressing the HPV16/18 E6/E7 genes were successfully constructed and propagated

**Figure 1:**
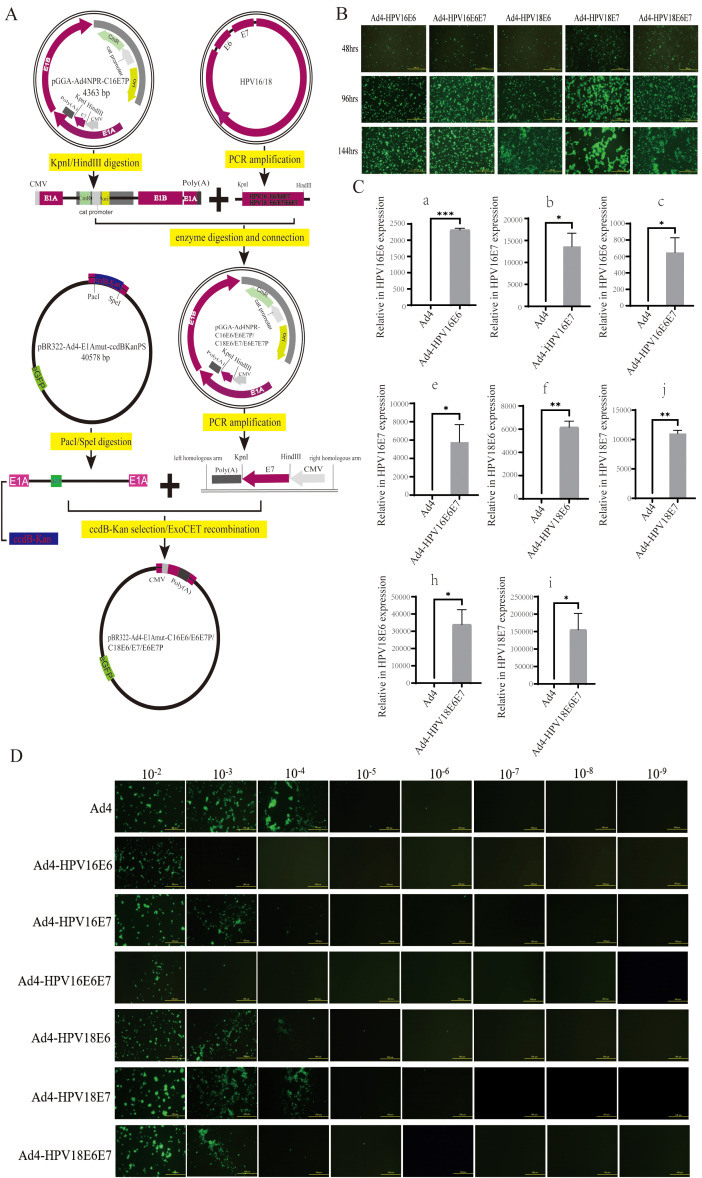
Generation of the recombinant viruses and evaluation of the HPV16/18 E6/E7 genes. **(A)** A schematic diagram for the construction of the recombinant virus plasmids PBR322-Ad4-E1Amut-C16E6P/C16E6E7P/C18E6P/C18E7P/C18E6E7P. **(B)** HEK293T cells transfected with pAd4-HPV16E6, pAd4-HPV16E6E7, pAd4-HPV18E6, pAd4-HPV18E7 and pAd4-HPV18E6E7 to obtain recombinant viruses. **(C)** RT-qPCR analysis revealing the HPV16/18 E6/E7/E6E7 genes exclusively detected in HEK293T cells after the infection of recombinant viruses. **(C-a)** Expression of the HPV16 E6 gene in cells infected with the recombinant virus Ad4-HPV16E6; **(C-b)** Expression of the HPV16 E7 gene in cells infected with the recombinant virus Ad4-HPV16E7; **(C-c)** Expression of the HPV16 E6 and E7 genes in cells infected with the recombinant virus Ad4-HPV16E6E7; **(C-d)** Expression of the HPV18 E6 gene in cells infected with the recombinant virus Ad4-HPV18E6; **(C-e)** Expression of the HPV18 E7 gene in cells infected with the recombinant virus Ad4-HPV18E7; **(C-f)**: Expression of the HPV18 *P < 0.05, **P < 0.01 and ****P < 0.0001 values were considered statistically significant.. **(D)** HEK293T cells infected with eight 10-fold serial dilutions of Ad4-HPV16E6, Ad4-HPV16E7, Ad4-HPV16E6E7, Ad4-HPV18E6, Ad4-HPV18E7 and Ad4-HPV18E6E7 and Ad4 viruses to calculate the viral titer.

The schematic diagram illustrating the specific construction of recombinant virus plasmids expressing HPV16/18 E6/E7 genes, including pBR322-Ad4-E1Amut-C16E6P, C16E6E7P, C18E6P, C18E7P, and C18E6E7P, is shown in **Figure 1A**. The construction and validation of recombinant adenoviral plasmids expressing HPV16/18 E6/E7 genes are depicted in **Supplementary Figure S1**. To generate the recombinant viruses Ad4-HPV16E6/E7 and Ad4-HPV18E6/E7/E6E7, the HEK293T cells were transfected with the recombinant virus genomes pAd4-HPV16E6/E7 and pAd4-HPV18E6/E7/E6E7 for 24-144 hours. The results indicated that the optimal infection duration was 96 hours (**Figure 1B**). At the 96-hour timepoint, the medium containing recombinant viruses was harvested, denoted as the P0 generation. Subsequently, HEK293T cells were reinfected with the P0 medium of Ad4-HPV16E6/E6E7 and Ad4-HPV18E6/E7/E6E7 for 96 hours, resulting in the production of the P1 viral-containing medium. This procedure was repeated until we obtained the P7 generation viral medium. To evaluate the stability of HPV16/18 E6/E7 gene transcription, we infected C33A cells with the recombinant viruses Ad4-HPV16/18E6/E7/E6E7 and subsequently detected the expression of the E6/E7 genes using qRT-PCR.

Our RT-qPCR analysis revealed that the presence of the HPV16 E6 gene was detected in the Ad4-HPV16E6 group, the HPV16 E7 gene was detected in the Ad4-HPV16E7 group, the HPV16 E6 and E7 genes were detected in the Ad4-HPV16E6E7 group, the HPV18 E6 gene was detected in the Ad4-HPV18E6 group, the HPV18 E7 gene was detected in the Ad4-HPV18E7 group, the HPV18 E6 and E7 genes were detected in the Ad4-HPV18E6E7 group, while no amplification signal was observed in the Ad4 group as expected (**Figure 1C**). These results indicate that the HPV16/18 E6/E7 genes of the recombinant viruses were effectively transcribed in cells. Virus titration was performed on Ad4 and the recombinant virus Ad4-HPV16E7 obtained in our laboratory, as well as the newly constructed recombinant viruses HPV16E6/E7 and Ad4-HPV18E6/E7/E6E7 (**Figure 1D**), following previously described methods (Lackner et al., 2022; Minarovits et al., 2016). The virus titer was determined to be TCID50 = 10^-6.5^/0.1ml (Ad4), TCID50 = 10^-4.6^/0.1ml (Ad4-HPV16E6), TCID50 = 10^-5.7^/0.1ml (Ad4-HPV16E7), TCID50 = 10^-4.6^/0.1ml (Ad4-HPV16E6E7), TCID50 = 10^-5.7^/0.1ml (Ad4-HPV18E6), TCID50 = 10^-5.9^/0.1ml (Ad4-HPV18E7), and TCID50 = 10^-5.3^/0.1ml (Ad4-HPV18E6E7), respectively. The above experimental results indicate that the recombinant viruses expressing the HPV16/18 E6/E7 genes were successfully constructed and propagated. To further investigate the role of E6/E7 genes in tumorigenesis, we will perform transcriptome sequencing on C33A cells infected with recombinant viruses expressing HPV16/18 E6/E7, in order to construct libraries and conduct biological analysis.

### 3.2 DEGs in C33A cells infected with the recombinant viruses Ad4-HPV16/18 E6/E7/E6E7 compared to that with Ad4

**Figure 2:**
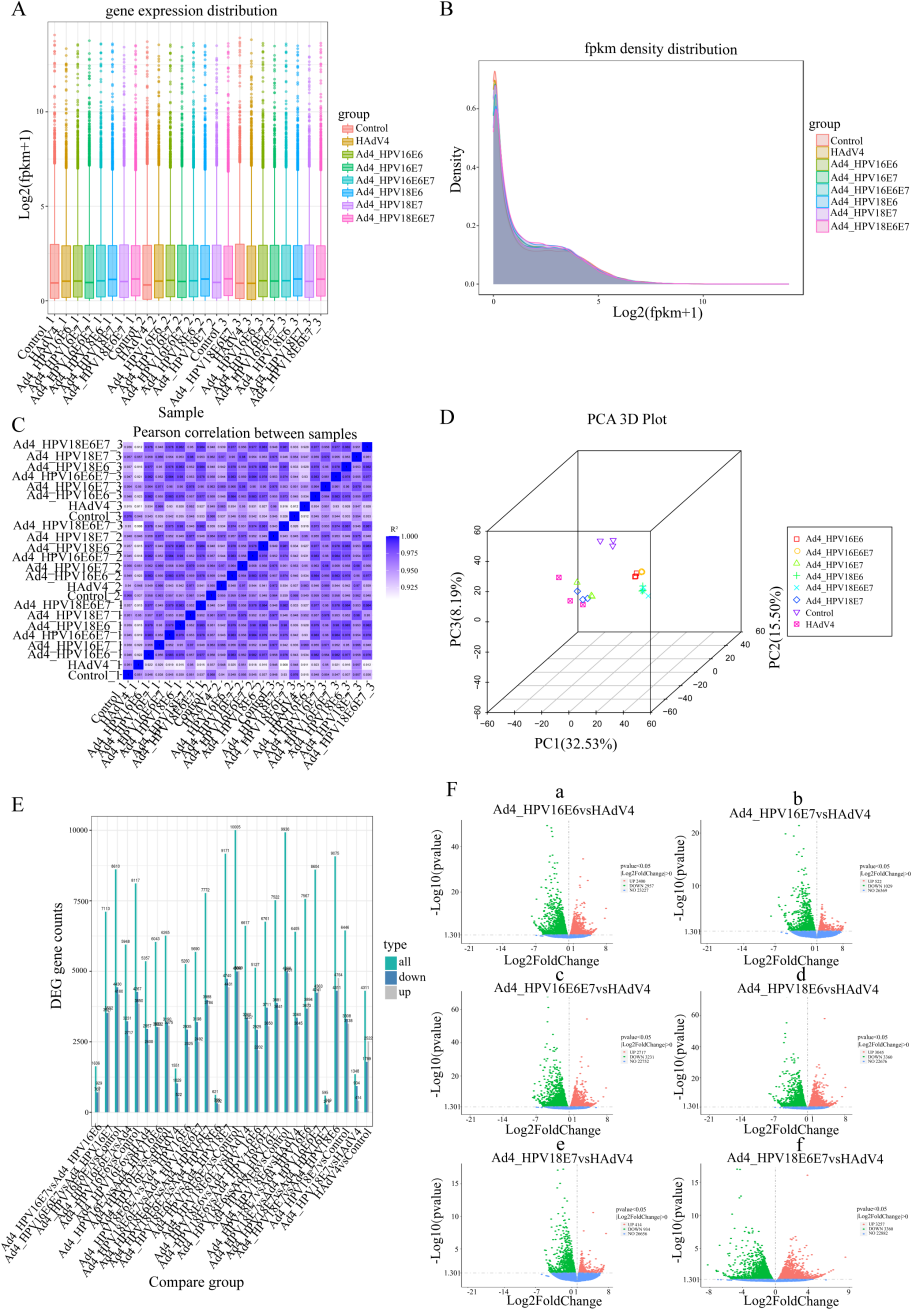
The comparative analysis of differentially expressed genes (DEGs). **(A)** Box plot of sample gene expression distribution. **(B)** Gene expression density diagram. The curves in different colors represent different samples; the abscissa indicates the log value of the FPKM and the ordinate indicates the probability density. **(C)** Heat map of correlation between samples. R^2^ >0.8 was considered a strong correlation. **(D)** PCA 3D plot. **(E)** Statistical histogram of the number of significantly DEGs in the different comparison combinations. **(F) (F-a)** Volcano map of DEGs between the Ad4-HPV16E6 and Ad4 grouup; **(F-b)** Volcano map of DEGs between the Ad4-HPV16E7 and Ad4 grouup; **(F-c)** Volcano map of DEGs between the Ad4-HPV16E6E7 and Ad4 grouup; **(F-d)** Volcano map of DEGs between the Ad4-HPV18E6 and Ad4 grouup; **(F-e)** Volcano map of DEGs between the Ad4-HPV18E7 and Ad4 grouup; **(F-f)** Volcano map of DEGs between the Ad4-HPV18E6E7 and Ad4 grouup.

We performed the RNA-Seq analysis to construct libraries and conduct biological analysis of all mRNA transcripts generated by C33A cells after infection with the recombinant viruses Ad4-HPV16/18 E6, E7, E6/E7. The expression values of all the genes (FPKM) in each sample were calculated, and box plots were used to visualize the distribution of gene expression levels in different samples. The box plot of gene expression distribution for each sample is shown in **Figure 2A**. The median gene expression levels across the samples are generally at the same level, indicating that the data can be directly used for subsequent differential analysis. The gene expression density diagram for the different samples indicated similar trends in gene abundance and gene expression density. Moreover, the log FPKM values were concentrated in the “0, 5” interval for all transcripts of the samples (**Figure 2B**). Moreover, the log FPKM values for all transcripts in the samples were concentrated in the “0, 5” interval (**Figure 2B**). This visually represents the gene expression levels and indicates that the RNA-Seq was successfully carried out. The correlation heatmap (**Figure 2C**) and three-dimensional principal component analysis (PCA) score plot (**Figure 2D**) showed similarities among replicates of the same sample and apparent differences among the different samples, indicating the reliability of the data. Subsequently, the comparative analysis of differentially expressed genes (DEGs) was conducted. The statistical histogram revealed that compared to the control group, the Ad4 group exhibited 4311 DEGs, the Ad4-HPV16E6 group exhibited 8117 DEGs, the Ad4-HPV16E7 group exhibited 6285 DEGs, the Ad4-HPV16E6E7 group exhibited 8610 DEGs, the Ad4-HPV18E6 group exhibited 9930 DEGs, the Ad4-HPV18E7 group exhibited 6446 DEGs, and the Ad4-HPV18E6E7 group exhibited 10005 DEGs (**Figure 2E**). To analyze the distribution of genes exhibiting significant expression variations between the Ad4-HPV16/18 E6, E7, E6/E7 group and the Ad4 group, we generated a volcano plot. The volcano plot (**Figure 2F**) illustrated that compared to the Ad4 group, there were 2400 upregulated genes and 2957 down-regulated genes in the Ad4-HPV16E6 group, 522 upregulated genes and 1029 down-regulated genes in the Ad4-HPV16E7 group, 2717 upregulated genes and 3231 down-regulated genes in the Ad4-HPV16E6E7 group, 3045 upregulated genes and 3360 down-regulated genes in the Ad4-HPV18E6 group, 414 upregulated genes and 934 down-regulated genes in the Ad4-HPV18E7 group, and 3257 upregulated genes and 3360 down-regulated genes in the Ad4-HPV18E6E7 group. This result suggests that the expression of HPV16/18 E6/E7 genes leads to differential gene expression in the genome of C33A cells. We need to further analyze the DEGs from the different comparison groups to identify gene functions and key biological pathways associated with HPV infection.

### 3.3 Identification of co-expressed DEGs related to HPV infection and DEGs related to cervical cancer in C33A cells infected with the six recombinant viruses

**Figure 3:**
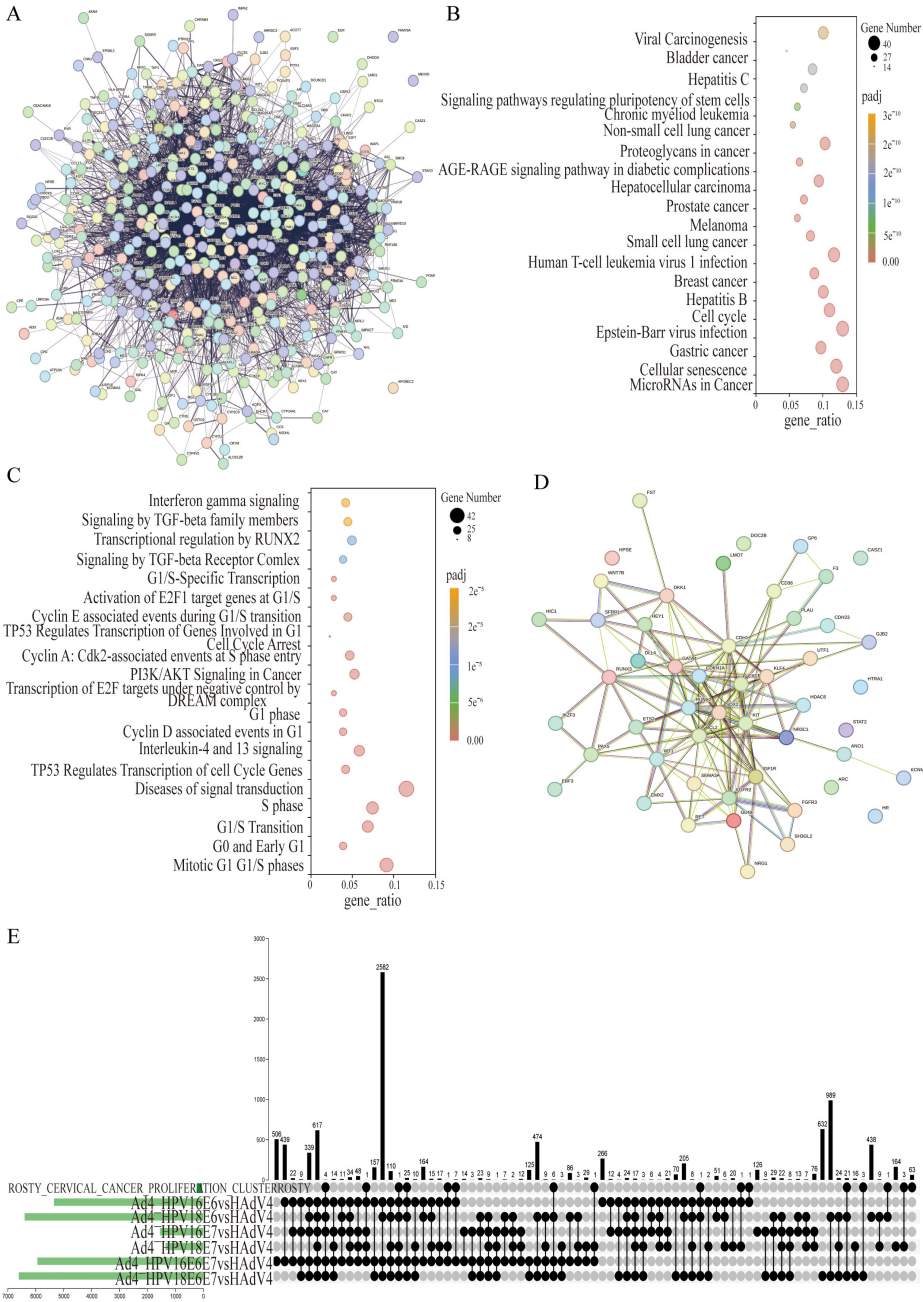
The co-expressed cervical cancer-related DEGs in the Ad4-HPV16/18 E6/E7/E6E7 group relative to the Ad4 group. **(A)** STRING protein–protein interaction of the 426 HPV-associated DEGs. **(B)** KEGG pathway analysis of 426 DEGs. **(C)** DisGeNET enrichment analysis of 426 DEGs. The size of the dots represents the number of genes annotated to the pathway, and the color from red to purple represents the significance level of pathway enrichment; the redder the color is, the more significant the result. **(D)** STRING protein–protein interaction of the 50 HPV-associated co-expressed DEGs in the Ad4-HPV16/18 E6, E7, E6/E7 group relative to the Ad4 group. Each node symbolizes the entirety of proteins generated by an individual gene locus responsible. Colored nodes are query proteins and the first shell of interactors. Edges represent protein-protein associations. Associations are meant to be specific and meaningful, i.e., proteins jointly contribute to a shared function. **(E)** UpSet diagram of the DEGs between the Ad4-HPV16/18 E6, E7, E6/E7 group relative to the Ad4 group and cervical cancer-related genes.

To identify genes associated with HPV infection, a comprehensive gene search was conducted using OMIM (https://www.ncbi.nlm.nih.gov/omim) and Genotype (https://www.ncbi.nlm.nih.gov/gap/phegeni), resulting in the identification of 418 genes related to HPV infection. PubMed (https://pubmed.ncbi.nlm.nih.gov) was extensively reviewed, yielding 1308 genes associated with HPV infection, and an additional 231 genes were obtained from the human papillomavirus infection pathway (hsa05165) in KEGG. These genes were then cross-screened with significantly different genes obtained from RNA-Seq sequencing results. After filtering, a total of 426 differentially expressed genes (DEGs) were identified as HPV infection-related. The filtered genes were further analyzed using the STRING database to construct a protein-protein interactions (PPIs) network. The resulting network consisted of 426 nodes, 5560 edges, and an expected number of 2922 edges. The average node degree was 26.1, the average local clustering coefficient was 0.476, and the PPI enrichment p-value was <1.0×10^-16^ (**Figure 3A**). This enrichment suggests a biologically interconnected nature of the proteins within the network as a collective entity.

We further analyzed 426 DEGs associated with HPV infection using KEGG and DisGeNET, revealing metabolic pathways and disease associations. The results were visualized through bubble diagrams, presenting the top enriched terms and pathways. The KEGG pathway analysis revealed that 426 DEGs are primarily involved in cancer-related pathways such as MicroRNAs and Cell cycle pathways (**Figure 3B**). Furthermore, the DisGeNET pathway analysis indicated these genes were mainly enriched in Mitotic G1 G1/S phases, Diseases of signal transduction, S phase, G1/S Transition, Interleukin-4 and 13 signaling, PI3K/AKT Signaling in Cancer, TP53 Regulates Transcription of Cell Cycle Genes, G1 phase, Activation of E2F1 target genes at G1/S, and Transcription of E2F targets under negative control by DREAM complex (**Figure 3C**). The enrichment analysis results indicate that 426 DEGs are involved in key pathways such as the cell cycle, PI3K-Akt signaling pathway, and TP53 regulation of cell cycle gene transcription in HPV-induced cancer. This provides data support for further research into the role of E6/E7 genes in tumorigenesis. We further screened 50 differentially expressed genes among these 426 genes, which were co-expressed in the Ad4-HPV16/18 E6/E7/E6E7 group. The filtered genes were further analyzed using the STRING database to construct a PPI network. The resulting network consisted of 50 nodes, 149 edges, and an expected number of 43 edges. The average node degree was 5.96, the average local clustering coefficient was 0.518, and the PPI enrichment p-value was <1.0×10^-16^ (**Figure 3D**). This enrichment suggests a biologically interconnected nature of the proteins within the network as a collective entity. An upset plot diagram was employed to intersect the seven gene sets (Cervical Cancer Proliferation Cluster and Ad4-HPV16/18 E6/E7/E6E7) and obtained 77 cervical cancer-related genes in the Ad4-HPV16/18 E6/E7/E6E7 group (**Figure 3E**). To further identify DEGs significantly expressed in cervical cancer related to E6/E7, the 50 DEGs co-expressed in the E6/E7 gene expression groups need to be subjected to clustering and interaction analysis along with the 77 genes associated with HPV infection-induced cervical cancer.

### 3.4 Cervical cancer-related up-regulated genes GP6, CD36, HDAC6, ESPL1, and DNMT3B among DEGs

**Figure 4:**
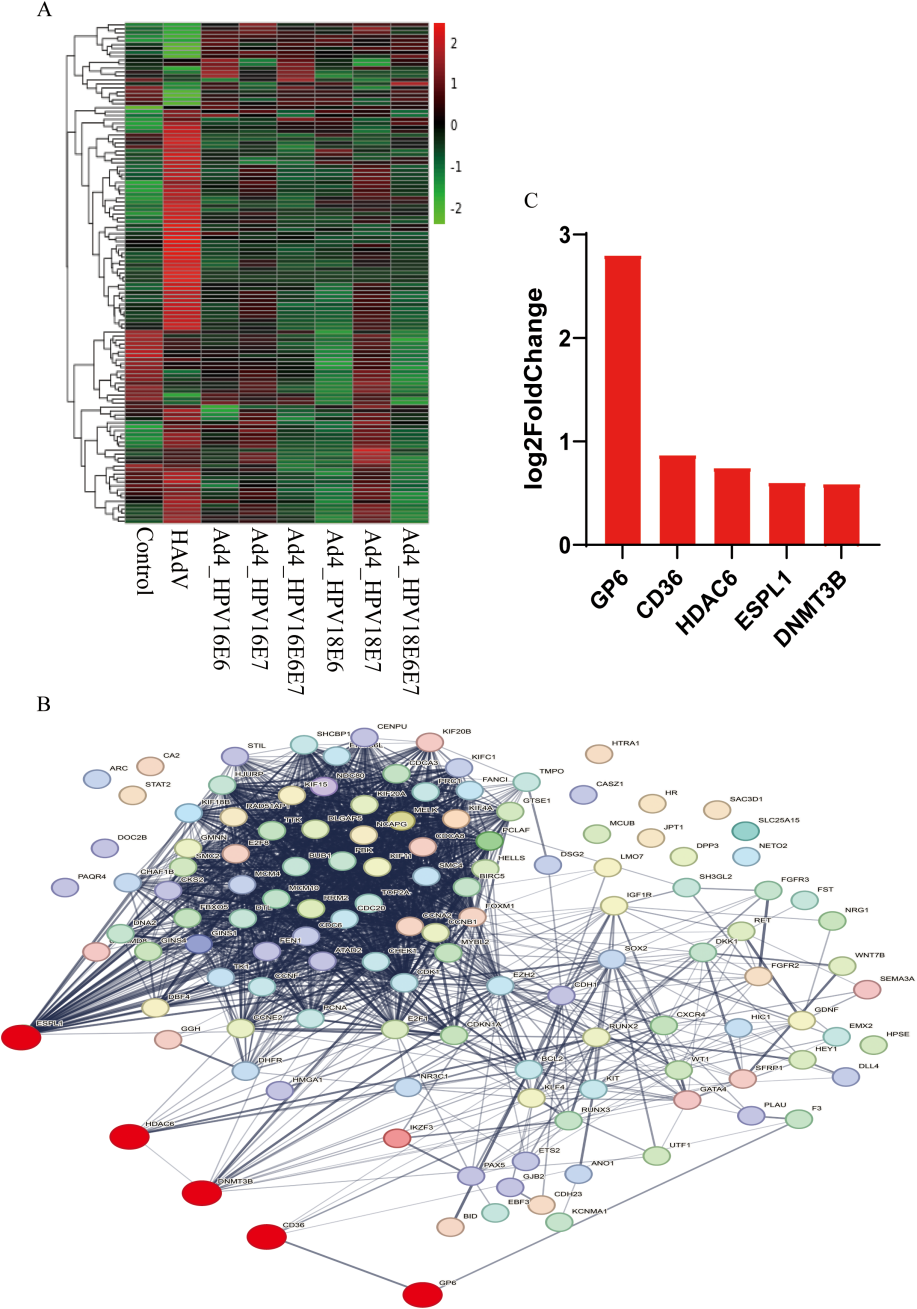
The relationship among GP6, CD36, HDAC6, ESPL1, DNMT3B, and oncogenic pathways in cervical cancer. **(A)** The heat map of the 50 HPV-associated co-expressed DEGs and 77 cervical cancer-related DEGs in the Ad4-HPV16/18 E6/E7/E6E7, Ad4, and control groups. The greener the color of the heatmap, the lower the gene expression, and the redder the color of the heatmap, the higher the gene expression. **(B)** STRING protein–protein interaction of the 50 HPV-associated co-expressed DEGs and 77 cervical cancer-related DEGs. **(C)** The five upregulated genes in the Ad4-HPV16/18 E6/E7/E6E7 group relative to the Ad4 group and the control group.

Cluster analysis was conducted on the expression levels of the 50 DEGs co-expressed in the Ad4-HPV16/18 E6/E7/E6E7 group and 77 common cervical cancer-related DEGs, as depicted in the heat map (**Figure 4A**). The 127 genes were further analyzed using the STRING database to construct a protein-protein interactions (PPIs) network. The resulting network consisted of 127 nodes, 1779 edges, and an expected number of 296 edges. These 1779 edges indicate 1779 associations, representing physical or functional interactions between all the proteins encoded by the 127 genes. The average node degree was 28, the average local clustering coefficient was 0.638, and the PPI enrichment p-value was <1.0×10^-16^. This enrichment suggests a biologically interconnected nature of the proteins within the network as a collective entity. In the PPIs network, the Red color indicates upregulated DEGs in the Ad4-HPV16/18 E6, E7, E6/E7 group (**Figure 4B**). Based on cluster analysis and the evidence of interactions among these DEGs provided by PPIs, we further screened five upregulated genes (GP6, CD36, HDAC6, ESPL1 and DNMT3B) in the Ad4-HPV16/18 E6/E7/E6E7 group that were expressed relative to the Ad4 and control groups (**Figure 4C**). Among these, HDAC6 and DNMT3B are key proteins involved in epigenetic modifications. This result suggests that E6/E7 may promote histone modification and DNA methylation by upregulating HDAC6 and DNMT3B. Therefore, we will conduct experiments and further analyses to validate the upregulation of HDAC6 and DNMT3B expression by E6/E7.

### 3.5 Recombinant viruses expressing E7 upregulate the DNMT3B and HDAC6 genes in C33A cells

**Figure 5:**
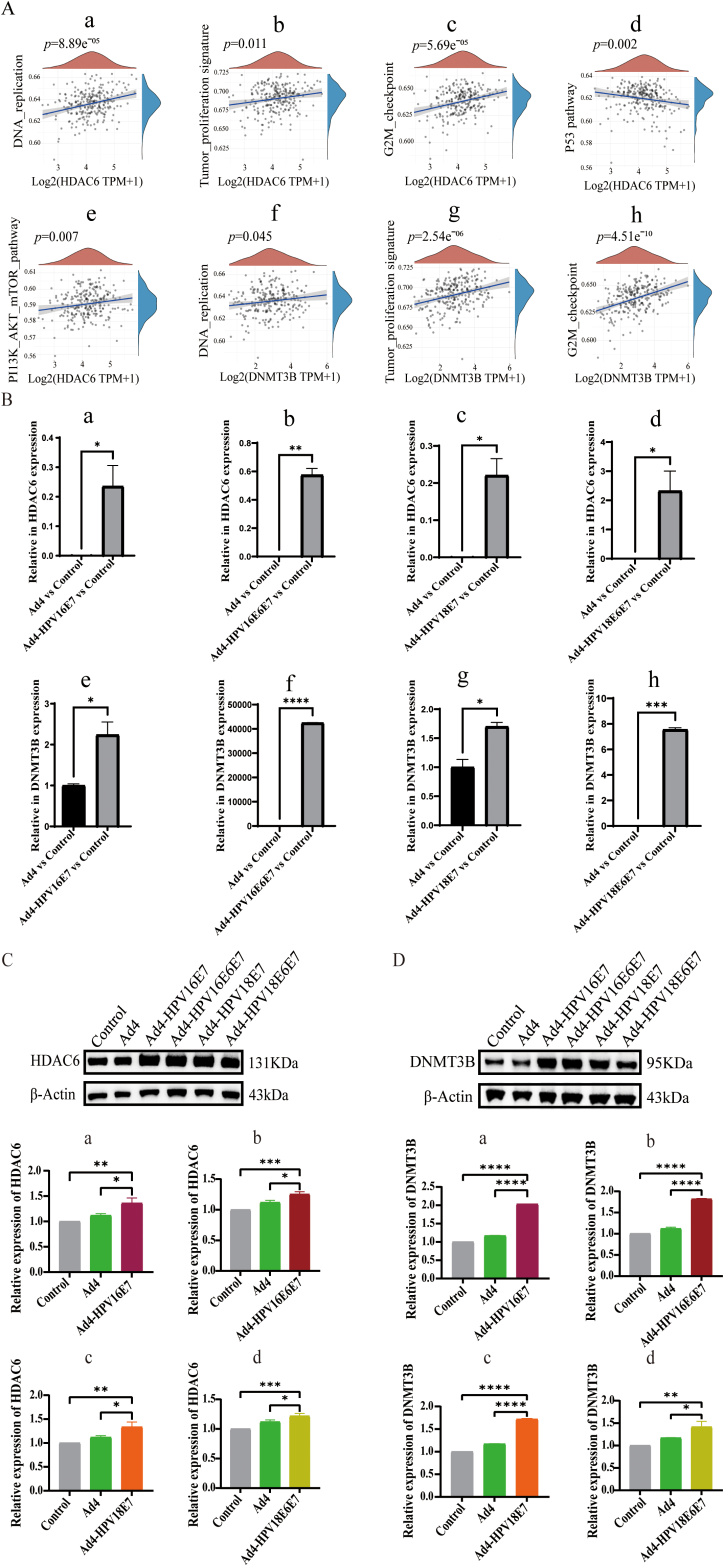
Recombinant viruses Ad4-HPV16/18E7/E6E7 induce upregulation of CD36 gene in C33A cells. **(A)** Spearman correlation analysis revealing the correlation between HDAC6 and DNMT3B and key pathways associated with cervical cancer. **(A a-e)** The significant correlations between HDAC6 and DNA replication, tumor proliferation signature, G2M checkpoint, p53 pathways, and PI3K/AKT/mTOR signaling pathways. **(A f-h)** The significant correlations between HDAC6 and DNA replication, tumor proliferation signature, and G2M checkpoint signaling pathways. *P* < 0.05 was considered significant. **(B)** qRT-PCR detection of the relative expression levels of HDAC6 and DNMT3B. **(B-a)** The relative expression levels of HDAC6 in Ad4-HPV16E7 and Ad4 group. ∗*P* < 0.05 compared with the Ad4 groups. **(B-b)** The relative expression levels of HDAC6 in Ad4-HPV16E6E7 and Ad4 group. ∗∗*P* < 0.01 compared with the control and Ad4 groups. **(B-c)** The relative expression levels of HDAC6 in Ad4-HPV18E7 and Ad4 group. ∗*P* < 0.05 compared with the control and Ad4 groups. **(B-d)** The relative expression levels of HDAC6 in Ad4-HPV18E6E7 and Ad4 group. ∗*P* < 0.05 compared with the control and Ad4 groups. **(B-e)** The relative expression levels of DNMT3B in Ad4-HPV16E7 and Ad4 group. ∗*P* < 0.05 compared with the Ad4 groups. **(B-f)** The relative expression levels of DNMT3B in Ad4-HPV16E6E7 and Ad4 group. ∗∗∗∗*P* < 0.0001 compared with the control and Ad4 groups. **(B-g)** The relative expression levels of DNMT3B in Ad4-HPV18E7 and Ad4 group. ∗*P* < 0.05 compared with the control and Ad4 groups. **(B-h)** The relative expression levels of DNMT3B in Ad4-HPV18E6/E7 and Ad4 group. ∗*P* < 0.05 compared with the control and Ad4 groups. **(C)** Representative images of Western blot relative expression of HDAC6 protein. **(C a-d)** Statistical analysis of Western blot results. *P < 0.05, **P < 0.01, and ***P < 0.001 values were considered statistically significant. **(D)** Representative images of Western blot relative expression of DNMT3B protein. **(D a-d)** Statistical analysis of Western blot results. *P < 0.05, **P < 0.01 and ****P < 0.0001 values were considered statistically significant.

To further analyze the regulatory effect of the E6/E7 genes on HDAC6, we searched the Kyoto Encyclopedia of Genes and Genomes (KEGG) database for pathways related to HPV infection in cervical cancer. In the HPV-induced cervical cancer pathway, we found that E7 directly interacts with HDAC6, contributing to cell transformation. Studies have found that DNMT3B mRNA transcripts are significantly overexpressed in the highly invasive cervical cancer cell lines HeLa and Caski (Su et al., 2013). It is well known that pathways such as DNA replication, tumor proliferation signature, G2M checkpoint, p53 pathways, and PI3K/AKT/mTOR signaling pathways are highly correlated with the occurrence and development of cervical cancer (Moody and Laimins, 2010; Bossler et al., 2019; Hussain et al., 2021). Therefore, we assembled a set of genes associated with these cervical cancer-related pathways and employed the ssGSEA algorithm to calculate the scores of the HDAC6 and DNMT3B genes in these pathways. Spearman correlation analysis represented the significant correlation between HDAC6 and DNA replication, tumor proliferation signature, G2M checkpoint, p53 pathways, and PI3K/AKT/mTOR signaling pathways. It also revealed the significant correlation between DNMT3B and DNA replication, tumor proliferation signature, and G2M checkpoint signaling pathways (**Figure 5A**). To further investigate the regulatory impact of the E7 virus on HDAC6 and DNMT3B in HPV-negative cervical cancer cells, C33A cells were separately infected with recombinant viruses expressing the E7 gene, including Ad4-HPV16E7, Ad4-HPV16E6E7, Ad4-HPV18E7, and Ad4-HPV18E6E7, while the Ad4-infected and non-infected C33A cells served as the control group. RNA was extracted from the cells, reverse transcribed into cDNA, and specific primers targeting HDAC6 were used for qRT-PCR. The qRT-PCR results showed that the expression of the HDAC6 and DNMT3B genes was upregulated in the Ad4-HPV16E7, Ad4-HPV16E6E7, Ad4-HPV18E7, Ad4-HPV18E6E7 groups compared to the Ad4 and control groups (**Figure 5B**). Western Blot analysis further confirmed that HDAC6 and DNMT3B protein expression increased in cells infected with Ad4-HPV16E7, Ad4-HPV16E6E7, Ad4-HPV18E7, and Ad4-HPV18E6E7 compared to Ad4-infected and uninfected cells (**Figures 5C, D**). The above results suggest that the E7 gene can upregulate the expression of HDAC6 and DNMT3B in C33A cells.

## 4 Discussion

HPV infection and its related diseases remain a major global health burden, with HPV being responsible for approximately 4.5% of the global cancer burden (Su et al., 2021; Marshall et al., 2022). It is well established that HPV is the etiological and biological carcinogen for HPV-related lesions and cancers (An et al., 2008; Su et al., 2021). Although prophylactic HPV vaccines are available, they do not have a strong therapeutic effect on pre-existing HPV infections and HPV-associated lesions (Guo et al., 2011). The oncogenicity of HPV is primarily dependent on the sustained expression and activity of the viral proteins E6 and E7, which alter interconnected cellular processes by interacting with over 100 different cellular proteins, thereby promoting tumorigenesis (Murthy et al., 2017; Dust et al., 2022). Some *in vitro* and xenograft studies have also indicated that cancer cells undergo senescence or apoptosis in the absence of E6 and E7 activity, thus demonstrating the absolute requirement of E6 and E7 for the sustained presence of HPV-mediated cancers (Soto et al., 2017; Pandey et al., 2021). The consistent expression of E6 and E7 viral proteins in HPV-related diseases and cancerous lesions makes them potential ideal targets for the development of therapeutic HPV vaccines (Zeng et al., 2011).

Isolating and culturing HPV *in vitro* is extremely difficult, highlighting the significance of constructing recombinant viruses that continuously express HPV E6/E7 genes *in vitro* for studying the role of E6/E7 in cancer development and treating HPV-related diseases (Wang et al., 2022). Thus, we established a platform of recombinant adenovirus vectors expressing the HPV16 E7 gene in our laboratory to construct recombinant virus particles that separately express HPV16/18 E6, HPV18 E7, and simultaneously express HPV16 E6 and E7, as well as HPV18 E6 and E7 genes. These recombinant viruses were employed to infect HEK293T cells, thereby achieving continuous expression of HPV16/18 E6/E7 genes in the cells.

The HPV E6/E7 oncoproteins disrupt cellular homeostasis by promoting proliferation, inhibiting apoptosis, and blocking epithelial differentiation, driving infected cells toward tumor progression (Nilsson et al., 2018; Das et al., 2020; Lou et al., 2022). However, the detailed carcinogenic mechanisms of E6 and E7 have not been fully elucidated, and there is currently no virus-specific therapy for HPV infection-related cancers (Dust et al., 2022; Young et al., 2022). Herein, we successfully constructed recombinant viruses expressing HPV16/18 E6/E7 genes to infect C33A cells to achieve continuous expression of HPV16/18 E6/E7 genes in cells. Total RNA was extracted from the cells and transcriptome sequencing was performed using RNA-Seq technology. Subsequent bioinformatics analysis identified significantly DEGs and the signaling pathways they are involved in related to HPV infection. The RNA-Seq results were cross-referenced with reliable sources such as OMIM, Genotype, KEGG, DisGeNET, and PubMed, resulting in the identification of 426 DEGs. Our KEGG pathway analysis showed that these DEGs are mainly involved in cancer-related pathways, such as MicroRNA in cancer and cell cycle pathways (**Figure 3B**). Additionally, our DisGeNET pathway analysis indicated that these genes are mainly enriched in mitotic G1 G1/S phase, signal transduction diseases, S phase, G1/S transition, interleukin-4 and 13 signaling, PI3K/AKT signaling in cancer, TP53-regulated cell cycle gene transcription, G1 phase, E2F1 target gene activation in G1/S phase, and E2F target gene transcription under DREAM complex negative control. Previous studies have confirmed that E6/E7 can promote carcinogenesis by regulating pathways involved in the cell cycle, cellular transformation, proliferation and differentiation, epigenetic modifications, DNA damage response, and genetic instability (Vats et al., 2021). However, the specific mechanisms by which E6/E7 influences the cell cycle and proliferation pathways remain unclear. Therefore, the pathway enrichment analysis results from this study provide a research direction for further exploring the roles of E6/E7 in these processes. We further screened 50 DEGs co-expressed in Ad4-HPV16/18 E6/E7 groups and 77 cervical cancer-related DEGs from these 426 genes. Expression levels of these 50 DEGs and 77 common cervical cancer-related DEGs were clustered and a protein-protein interaction network was constructed. Five upregulated genes (GP6, CD36, HDAC6, ESPL1, and DNMT3B) were further identified in the Ad4-HPV16/18 E6/E7 group relative to the Ad4 and control groups.

Among these, the upregulated gene, the HDAC6, a member of the HDAC family, is involved in cell survival, cell movement, cell cycle progression, protein degradation, and developmental events and has become a promising target for managing various human diseases, including cancer (Lin et al., 2009; Banerjee et al., 2018; Ling et al., 2020). Previous studies have identified HDAC6 as a candidate gene associated with HPV-positive expression in cervical cancer (Lourenço de Freitas et al., 2020). Although the oncogenic role of HDAC6 has been reported in cervical cancer, its mechanism in the development of HPV-positive cervical cancer remains unclear (Luczak and Jagodzinski, 2008). Moreover, the heterogeneity of cervical epithelial cells in HPV-induced cervical cancer remains largely unknown (Sancakli Usta et al., 2020). Cervical cancer cells are addicted to the expression of the HPV oncoprotein E7, whereas the E7 oncoprotein acts in part by co-opting HDAC (Huang et al., 2015). We found that in the viral carcinogenesis pathway, E7 directly acts on HDAC6 to induce cell transformation in HPV infection-related cancers (cervical cancer, anal cancer, penile cancer, and head and neck carcinoma), inducing the malignant transformation of cells. Several studies have shown that HDAC6 also plays a crucial role in the G0 or G1 cell cycle phases and can participate in cancer-related signaling pathways by regulating the cell cycle, apoptosis, and cell transformation (Shi et al., 2021).

Some studies have found that DNMT3B mRNA transcripts are significantly overexpressed in the highly invasive cervical cancer cell lines HeLa and Caski (Su et al., 2013). Additionally, research on the genomic profiles of HPV-immortalized keratinocyte cell lines has revealed elevated mRNA expression of DNMT3B (Wilting et al., 2006). Moreover, DNA methylation has been reported to directly complement HPV screening, serving as a molecular diagnostic and prognostic biomarker for cervical intraepithelial neoplasia and cervical cancer (Han et al., 2023). Therefore, we collected a set of genes related to cervical cancer-associated pathways and used the ssGSEA algorithm to calculate the scores of the HDAC6 and DNMT3B genes in these pathways. Our Spearman correlation analysis reveals significant correlations between HDAC6 and key pathways, including DNA replication, tumor proliferation signature, G2M checkpoint, p53 pathways, and PI3K/AKT/mTOR signaling pathways, as well as between DNMT3B and DNA replication, tumor proliferation signature, and G2M checkpoint signaling pathways, revealing the potential role of the HDAC6 gene in HPV infection-related cervical cancer.

To further investigate the regulatory effect of the E7 gene on HDAC6 and DNMT3B in HPV-negative cervical cancer cells, we infected C33A cells with recombinant viruses expressing the E7 gene (Ad4-HPV16E7, Ad4-HPV16E6E7, Ad4-HPV18E7, and Ad4-HPV18E6E7). The qRT-PCR results indicated that compared with the Ad4 group and the control group, HDAC6 and DNMT3B expression was upregulated in the Ad4-HPV16E7, Ad4-HPV16E6E7, Ad4-HPV18E7, and Ad4-HPV18E6E7 groups. Western Blot results further confirmed that HDAC6 and DNMT3B protein expression increased in cells infected with Ad4-HPV16E7, Ad4-HPV16E6E7, Ad4-HPV18E7, and Ad4-HPV18E6E7 compared to Ad4-infected and uninfected cells. These findings suggest that recombinant viruses expressing HPV16/18 E7 can upregulate HDAC6 and DNMT3B gene expression in C33A cells. Our results indicate that alteration in HDAC6 and DNMT3B expression is associated with high-risk human papillomavirus infection and could promote the occurrence and development of cervical cancer. Therefore, this study provides important insights for further research on the molecular mechanisms of HPV infection, cervical cancer development, and potential therapeutic targets.

## Data Availability

The data presented in the study are deposited in NCBI (National Coalition Building Institute) SRA (Sequence Read Archive) database with the BioProject accession numbers PRJNA1163737.
