# Supplementary material for: Recombinant adenoviruses expressing HPV16/18 E7 upregulate the HDAC6 and DNMT3B genes in C33A cells
Source: Front Cell Infect Microbiol. 2024 Oct 1;14:1459572. doi: 10.3389/fcimb.2024.1459572 (PMC11473514; doi:10.3389/fcimb.2024.1459572)
Supplement: Supplementary file 1 [file DataSheet1.docx]

**Supplementary Material**

**Supplementary** **Table1. All *Escherichia coli* strains and plasmids evaluated in this study.**

| Strains/ Reference/Source | Genotype/Description | Reference/Source |
| --- | --- | --- |
| Strains |  |  |
| *E. coli* GB08-Red | *E. coli* strain harboring an arabinose inducible γβαA operon (redγ, redβ, redα and recA) at the ybcC locus | [1] |
| *E. coli* GB05-dir | derived by integrating the PBAD-ETgA operon into the ybcC locus in GB2005 | [2] |
| Plasmids |  |  |
| Ad4 | GenBank accession no. AY594253 | [3] |
| pGGA-Ad4NPR-C16E7P | Containing the Ad4 E1A and HPV16E7 genes | [4] |
| HPV16 | NCBI Reference Sequence: NC_001526.4 | [5] |
| HPV18 | GenBank accession no.  GCF_000865665.1 | This study |
| pBR322-Ad4-E1Amut-ccdBKanPS | Containing the Ad4 E1A and ccdB genes | [4] |


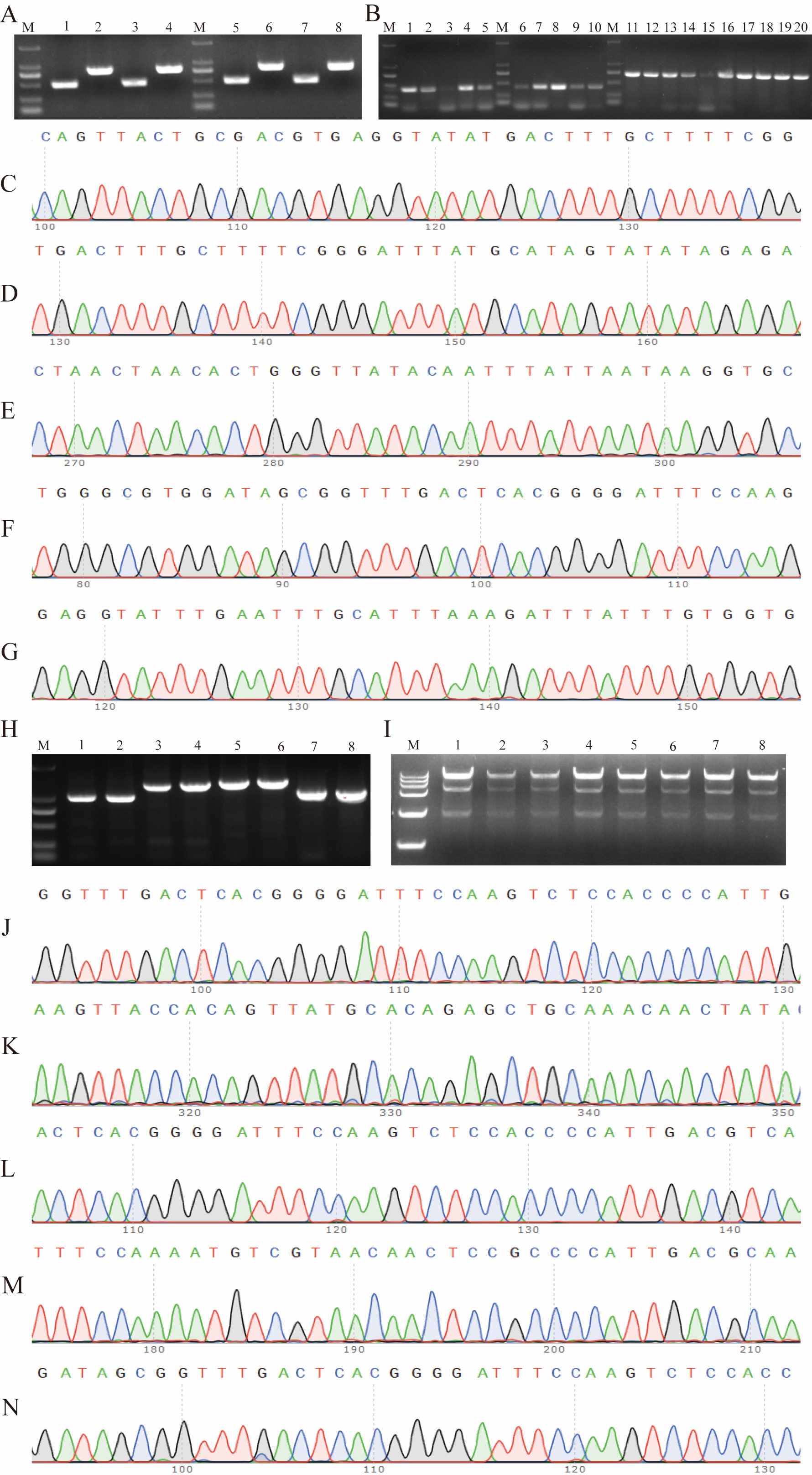


**Supplementary Figure 1. Construction and validation of recombinant adenovirus plasmids expressing HPV16/18 E6/E7 genes.** **A**: The DNA fragments of HPV16 E6/E6E7 and HPV18 E6/E7/E6E7. M: Mark DL2000; 1: The DNA fragments of HPV16 E6; 2,4: The DNA fragments of HPV16 E6E7; 3: The DNA fragments of HPV18 E6; 5,7: The DNA fragments of HPV18 E7; 6,8: The DNA fragments of HPV18 E6E7. **B**: Identification of the intermediate plasmids. 1-5: The HPV16 E6 DNA fragment amplified by PCR from the intermediate plasmid pGGA-Ad4NPR-C16E6P; 6,7: The HPV18 E6 DNA fragment amplified by PCR from the intermediate plasmid pGGA-Ad4NPR-C18E6P; 8-10: The HPV18 E7 DNA fragment amplified by PCR from the intermediate plasmid pGGA-Ad4NPR-C18E7P; 11-15: The HPV16 E6E7 DNA fragment amplified by PCR from the intermediate plasmid pGGA-Ad4NPR-C16E6E7P; 16-20: The HPV18 E6E7 DNA fragment amplified by PCR from the intermediate plasmid pGGA-Ad4NPR-C18E6E7P. **C**: The sequencing results of the intermediate plasmid pGGA-Ad4NPR-C16E6P. **D**: The sequencing results of the intermediate plasmid pGGA-Ad4NPR-C16E6E7P. **E**: The sequencing results of the intermediate plasmid pGGA-Ad4NPR-C18E6P. **F**: The sequencing results of the intermediate plasmid pGGA-Ad4NPR-C18E7P. **G**: The sequencing results of the intermediate plasmid pGGA-Ad4NPR-C18E6E7P. **H**: HPV16 E6/E6E7 and HPV18 E6/E7/E6E7 DNA fragments carrying Ad4 E1A homologous arms. M: Mark DL2000; 1,2: HPV16 E6 DNA fragments carrying Ad4 E1A homologous arms; 3,4: HPV16 E6E7 DNA fragments carrying Ad4 E1A homologous arms; 5,6: HPV18 E6E7 DNA fragments carrying Ad4 E1A homologous arms; 7: HPV18 E6 DNA fragments carrying Ad4 E1A homologous arms; 8: HPV18 E7 DNA fragments carrying Ad4 E1A homologous arms. **I**: Enzyme digestion verification of the recombinant adenovirus plasmids. M: Mark DL10000; 1,2: The *HindⅢ* enzyme digestion product of pBR322-Ad4-E1Amut-C16E6P; 3,4: The *HindⅢ* enzyme digestion product of pBR322-Ad4-E1Amut-C16E6E7P; 5,6: The *HindⅢ* enzyme digestion product of pBR322-Ad4-E1Amut-C18E6P; 7,8: The *HindⅢ* enzyme digestion product of pBR322-Ad4-E1Amut-C18E6E7P. **J**: The sequencing results of the recombinant adenovirus plasmids pBR322-Ad4-E1Amut-C16E6P. **K**: The sequencing results of the recombinant adenovirus plasmids pBR322-Ad4-E1Amut-C16E6E7P. **L**: The sequencing results of the recombinant adenovirus plasmids pBR322-Ad4-E1Amut-C18E6P. **M**: The sequencing results of the recombinant adenovirus plasmids pBR322-Ad4-E1Amut-C18E7P. **N**: The sequencing results of the recombinant adenovirus plasmids pBR322-Ad4-E1Amut-C18E6E7P.

**Reference**

[1] J. Fu, M. Teucher, K. Anastassiadis, W. Skarnes and A. F. Stewart A recombineering pipeline to make conditional targeting constructs. Methods in enzymology. 477(2010)125-144. 10.1016/s0076-6879(10)77008-7.

[2] J. Fu, X. Bian, S. Hu, H. Wang, F. Huang, P. M. Seibert, A. Plaza, L. Xia, R. Müller, A. F. Stewart, et al. Full-length RecE enhances linear-linear homologous recombination and facilitates direct cloning for bioprospecting. Nature biotechnology. 30(2012)440-446. 10.1038/nbt.2183.

[3] B. Lin, Z. Wang, G. J. Vora, J. A. Thornton, J. M. Schnur, D. C. Thach, K. M. Blaney, A. G. Ligler, A. P. Malanoski, J. Santiago, et al. Broad-spectrum respiratory tract pathogen identification using resequencing DNA microarrays. Genome research. 16(2006)527-535. 10.1101/gr.4337206.

[4] Y. Shao, P. Wang, Y. Zheng, H. Cui, Z. Lou, S. Li, F. Huang and C. Wu A replicative recombinant HPV16 E7 expression virus upregulates CD36 in C33A cells. Front. Microbiol. 14(2023)1259510. 10.3389/fmicb.2023.1259510.

[5] Q. Liu, Q. Shen, X. Bian, H. Chen, J. Fu, H. Wang, P. Lei, Z. Guo, W. Chen, D. Li, et al. Simple and rapid direct cloning and heterologous expression of natural product biosynthetic gene cluster in Bacillus subtilis via Red/ET recombineering. Scientific reports. 6(2016)34623. 10.1038/srep34623.
